# Involvement of orexin neurons in fasting- and central adenosine-induced hypothermia

**DOI:** 10.1038/s41598-018-21252-w

**Published:** 2018-02-09

**Authors:** Takahiro Futatsuki, Akira Yamashita, Khairunnisa Novita Ikbar, Akihiro Yamanaka, Kazunori Arita, Yasuyuki Kakihana, Tomoyuki Kuwaki

**Affiliations:** 1 Department of Physiology, Kagoshima University Graduate School of Medical & Dental Sciences, Kagoshima, 890-8544 Japan; 2 Department of Emergency and Intensive Care Medicine, Kagoshima University Graduate School of Medical & Dental Sciences, Kagoshima, 890-8544 Japan; 3 Department of Neurosurgery, Kagoshima University Graduate School of Medical & Dental Sciences, Kagoshima, 890-8544 Japan; 4 Research Institute of Environmental Medicine, Nagoya University, Nagoya, 464-8601 Japan

## Abstract

We examined whether orexin neurons might play a protective role against fasting- and adenosine-induced hypothermia. We first measured body temperature (BT) in orexin neuron-ablated (ORX-AB) mice and wild-type (WT) controls during 24 hours of fasting. As expected, the magnitude of BT drop and the length of time suffering from hypothermia were greater in ORX-AB mice than in WT mice. Orexin neurons were active just before onset of hypothermia and during the recovery period as revealed by calcium imaging *in vivo* using G-CaMP. We next examined adenosine-induced hypothermia via an intracerebroventricular administration of an adenosine A1 receptor agonist, N6-cyclohexyladenosine (CHA), which induced hypothermia in both ORX-AB and WT mice. The dose of CHA required to initiate a hypothermic response in ORX-AB mice was more than 10 times larger than the dose for WT mice. Once hypothermia was established, the recovery was seemingly slower in ORX-AB mice. Activation of orexin neurons during the recovery phase was confirmed by immunohistochemistry for c-Fos. We propose that orexin neurons play dual roles (enhancer in the induction phase and compensator during the recovery phase) in adenosine-induced hypothermia and a protective/compensatory role in fasting-induced hypothermia.

## Introduction

Some animals, especially small mammals living in cold climates, hibernate during the winter season. During hibernation, their metabolism decreases to be less than 10% of euthermic levels and their body temperature lowers to a level slightly higher than the environmental temperature, which is typically around 2–10 °C^1^. Although laboratory rats and mice do not hibernate, they undergo daily torpor, a mild form of hibernation^2,3^. During both hibernation and torpor, body temperature decreases cyclically several times. However, during torpor the cycles are shorter (minutes ~ hour) and have a higher minimum body temperature (~30 °C) when compared to the length (hours ~ days) and the body temperature (2–10 °C) of the cycles in hibernation. Although a common mechanism is presumed to exist for both hibernation and torpor, specifically in regards to active suppression of heat production and low set point of body temperature^4^, details have yet to be elucidated.

Although the regulatory mechanisms behind changes in body temperature during hibernation/torpor are not fully understood, medical doctors will often induce hypothermia for clinical purposes, which is effective for reducing brain damage after cardiac arrest and brain injury^5^. Reduction of temperature is also useful for long-term preservation of tissues and organs for transplantation^6^. At present, however, we only know how to cool the body to passively reduce metabolism and are not able to decrease it actively^7,8^. If the mechanisms underlying how hibernating animals decrease their body temperature and recover from hypothermia become fully understood, then the methods for inducing therapeutic hypothermia will improve.

Laboratory mice are useful for studying the brain mechanisms behind active hypothermia because genetically modified mice are widely available and torpor is easily observable under fasting conditions and/or via injection of various drugs, including adenosine (presumed metabolic signal of short energy supply) into the brain^9,10^. We have previously shown that hypothalamic orexin neurons contribute to stress-induced thermogenesis through activation of beta-3 receptors probably on the sympathetic nerves controlling the brown adipose tissue^11^. They also protect against hypothermia induced by cold environments^12^ and isoflurane anesthesia^13^. Orexin neuronal activity has also been shown to be inhibited by glucose^14^, leptin^14^, adenosine^15^, and hyperthermia^16^ at least *in vitro*. These findings prompted us to hypothesize that orexin neurons may also play a protective role against fasting- and adenosine-induced hypothermia. We also examined possible changes in the activity of melanin concentrating hormone (MCH)-containing neurons during central adenosine induced hypothermia. MCH neurons are located nearby orexin neurons^17^ and implicated in body temperature regulation because central infusion of MCH decrease body temperature^18^ and both fasting^19^ and cold exposure^20^ increases MCH in the hypothalamus.

## Results

### Fasting-induced hypothermia was more severe and longer lasting in ORX-AB mice

We have previously shown that orexin neurons contribute to stress-induced thermogenesis^11^ and protect against hypothermia induced by a cold environment^12^ and against hypothermia caused by isoflurane-anesthesia^13^. Here we examined whether orexin neurons also play a protective role against fasting-induced hypothermia.

**Figure 1:**
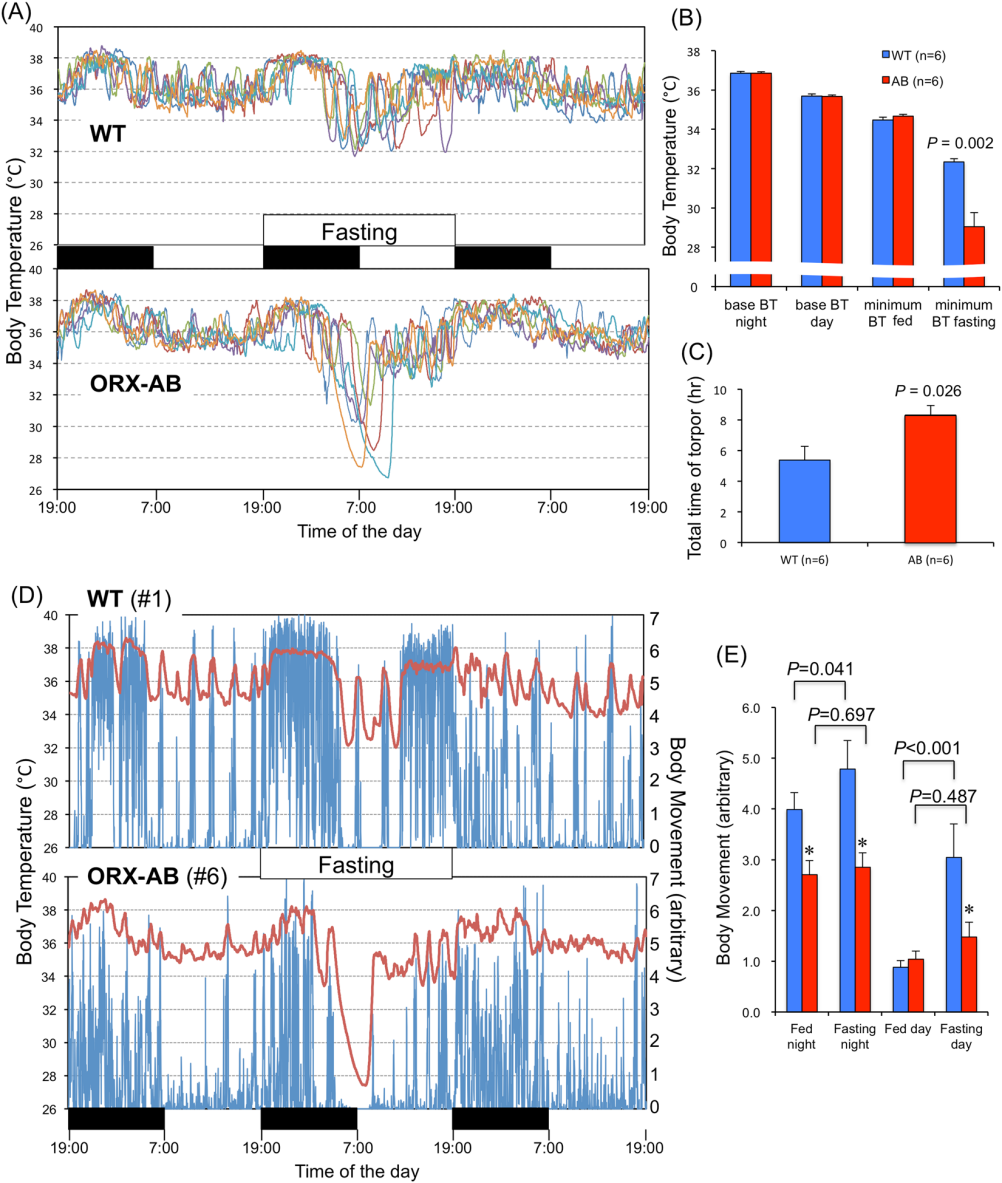
Fasting-induced hypothermia. (**A**) Individual traces of body temperature for three successive days are presented in different colors for wild-type (WT) mice and orexin neuron-ablated (ORX-AB) mice. Black and white bars in the middle indicate night and day, respectively. Food, but not water, was deprived for 24 hr for the second day. (**B**) Average body temperature (BT) for the night and day of the 1st day, minimum BT during the 1st day, and minimum BT during the fasting period. (**C**) Total time spent in hypothermia (torpor). (**D**) Representative tracings of body temperature (red, left ordinate) and body movement (blue, right ordinate) from a WT and an ORX-AB mouse. (**E**) Average body movement during the night and day in the 1st fed day and 2nd fasting day. In (**B**), (**C**) and (**E**), data are presented as mean ± SEM of the WT mice (n = 6) and ORX-AB mice (n = 6). P value was calculated using the Mann-Whitney U-test or two-way (genotypes x times) ANOVA with post-hoc Holm-Sidak multiple comparison test.

There were no differences in average body temperatures during both night and day between the WT and ORX-AB mice before food deprivation (Fig. 1A and B). Minimum body temperatures during the fed period were also not different between the two groups (Fig. 1B). Food deprivation induced repetitive hypothermic episodes (torpors) in all the tested WT and ORX-AB mice (Fig. 1A). Although there are apparent similarities between the WT and ORX-AB mice, detailed analyses showed quantitative differences. First, the minimum body temperature during the fasting period was significantly lower in ORX-AB mice (29.0 ± 0.7 °C, n = 6) than in WT mice (32.3 ± 0.2, n = 6, p = 0.002). In fact, the body temperature of WT mice never fell below 31.5 °C whereas that of ORX-AB mice did (Fig. 1A). Second, the onset of first torpor was significantly faster in ORX-AB mice (8.0 ± 0.4 hr after the start of fasting) than in WT mice (10.0 ± 0.5 hr, p = 0.026). Third, the number of torpors was significantly higher in ORX-AB mice (5.5 ± 0.6) than in WT mice (3.3 ± 0.6, p = 0.046). Lastly, the total time spent in torpor was significantly longer in ORX-AB mice (8.3 ± 0.6 hr) than in WT mice (5.4 ± 0.9, p = 0.026) (Fig. 1C).

Changes in body temperature closely coincided with changes in body movement during both fasting and fed periods (Fig. 1D,E). Of particular interest was that hypothermia was preceded by a period of vigorous movement, especially for the first hypothermic episode. In WT mice, fasting induced significant increase in body movement, presumably for food seeking, during both night and day (Fig. 1E). However, ORX-AB mice did not show such an increase during fasting. These data collectively support the notion that orexin neurons play a protective role against fasting-induced hypothermia.

### Orexin neuronal activity was high during the active period preceding the hypothermic episode and during the following recovery period

**Figure 2:**
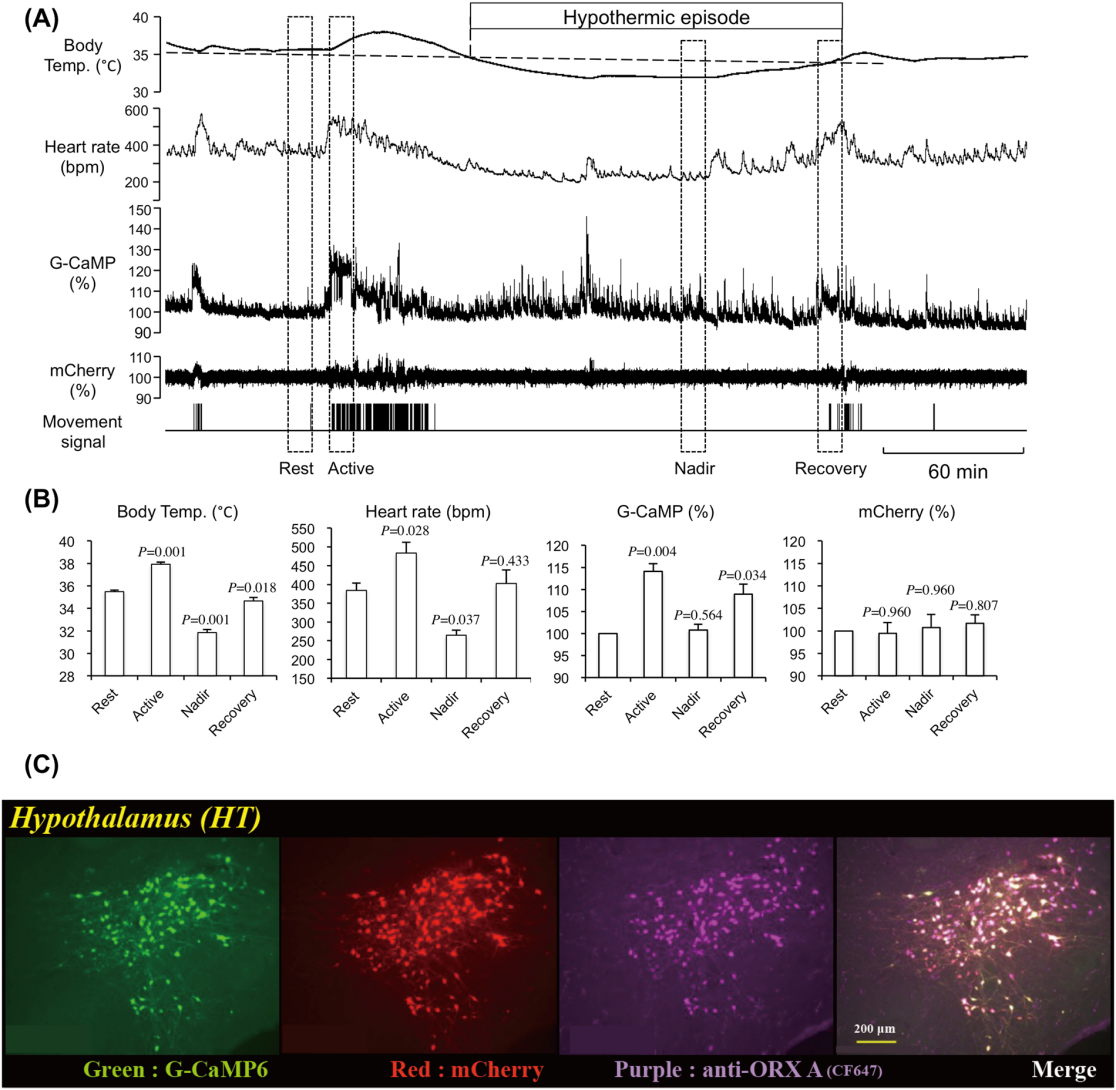
Activity recording of orexin neurons using fiber photometry during fasting-induced hypothermia. (**A**) Representative traces of body temperature, heart rate, G-CaMP fluorescence, mCherry fluorescence, and body movement in a mouse expressing G-CaMP and mCherry exclusively in orexin neurons. The first hypothermic episode during the fasting period is shown. (**B**) Averaged body temperature, heart rate, G-CaMP fluorescence, and mCherry fluorescence during four typical periods indicated by the dashed rectangles (10 min length) shown in A. See text for the definitions of Rest, Active, Nadir, and Recovery. Fluorescence intensity during the rest period was defined as 100%. (**C**) Histological evidence showing that G-CaMP and mCherry were exclusively expressed in almost all orexin neurons. In (**B**), data are presented as mean ± SEM of 5 animals. Data were collected only for the 1st hypothermic episode even if the animal experienced multiple hypothermic episodes. P values were calculated using the Holm-Sidak multiple comparison test.

Increased movement before and after the hypothermic episode (Fig. 1D) indicated possible activation of the orexin neurons during these periods. To test this possibility, we recorded the activity of orexin neurons using fiber photometry during fasting-induced hypothermia. Simultaneous recordings of G-CaMP fluorescence and mCherry fluorescence (Fig. 2A,C) gave us confidence that the recorded changes in G-CaMP fluorescence came from the changes in orexin neuronal activity because even though changes in C-CaMP fluorescence occasionally coincided with the changes in animal’s movement and/or heart rate, the associated changes in mCherry fluorescence were so small that movement/cardiovascular-associated noise in G-CaMP fluorescence should be minimal or nonexistent. In this experiment, G-CaMP fluorescence from 5 animals was averaged during 4 characteristic time windows because the length of an individual hypothermic episode varies from mouse to mouse and even varies among multiple episodes in a single mouse (Fig. 1A). The 4 time windows used were: Rest, the resting period before the hypothermia; Active, the active period preceding the hypothermic episode when the animal’s movement and heart rate increased; Nadir, the period when the body temperature during the hypothermic episode reaches nadir; and Recovery, just before the end of a hypothermic episode. As expected, increases in G-CaMP fluorescence, but not mCherry florescence, closely coincided with increases in body temperature, heart rate, and movement and were higher during the active period and the recovery period than during the resting period (Fig. 2B). Although body temperature and heart rate were significantly lower in the nadir period than the resting period, G-CaMP fluorescence during the nadir period was not different from the resting period. We cannot claim a causative relationship between orexin neuronal activity and body temperature, but these results are compatible with the notion that orexin neurons play a protective role against fasting-induced hypothermia.

### ORX-AB mouse was tolerant to central adenosine A1 receptor agonist-induced hypothermia

We next examined whether the orexin neurons play a similar protective role in another hypothermia model induced by an injection of an adenosine A1 receptor agonist.

**Figure 3:**
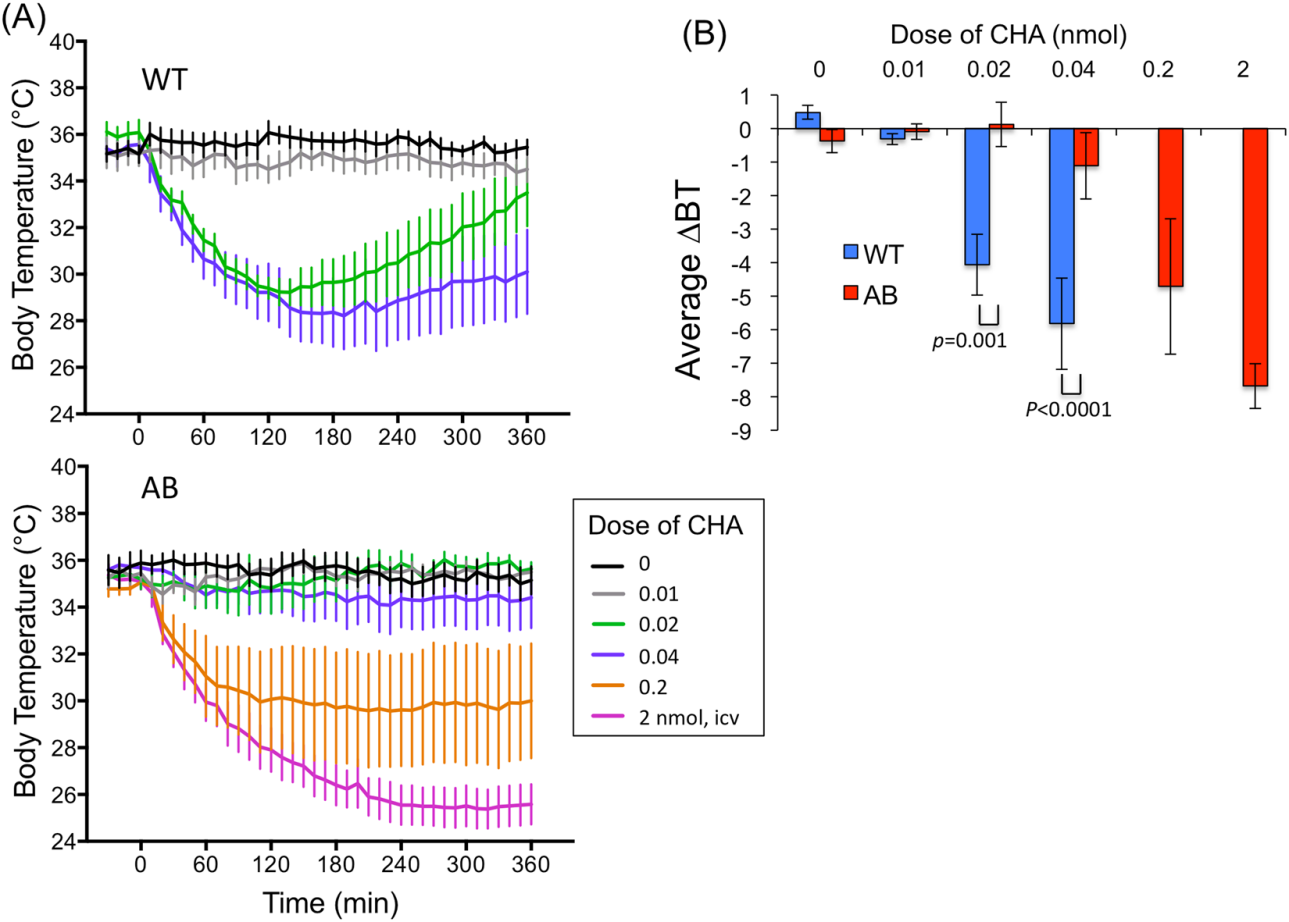
Effect of central administration of an adenosine A1 receptor agonist, N6-cyclohexyladenosine (CHA), on body temperature. (**A**) Time-related changes in body temperature in wild-type mice (WT) and orexin neuron-ablated mice (AB). CHA was intracerebroventricularly injected at time 0. (**B**) Changes in body temperature (BT) averaged for 6 hours following the injection. 2-way ANOVA indicated there were statistical difference between genotypes (F_1,40_ = 15.68, p = 0.0003) and doses (F_3,40_ = 10.00, p < 0.0001). P values were calculated using the Holm-Sidak multiple comparison test. Each point represents mean ± SEM from 6 animals.

In WT mice, I.C.V injection of an adenosine A1 receptor agonist, CHA (0.02–0.04 nmol), induced an abrupt and long lasting (>6 hr) decrease in body temperature with its nadir around 2–3 hr post-injection (Fig. 3A). Although the average changes in body temperature during the 6 hr of the observation period showed dose-dependency (F_3,40_ = 10.00, p < 0.0001) (Fig. 3B), the induction phase seemed to be an all-or-none type response pattern (Fig. 3A). WT mice were unable to tolerate higher doses (0.2 nmol) of CHA in our pilot study (n = 2) and therefore increased doses were not examined in those mice.

Contrary to our expectation, the same dose of CHA (0.02–0.04 nmol) that induced hypothermia in WT mice failed to do so in ORX-AB mice (Fig. 3). They tolerated the higher doses of CHA (0.2–2 nmol) that the WT mice could not and showed long lasting (>6 hr) hypothermia. Their body temperature had recovered by the next day of experimentation.

These data point out that a possible role for orexin neurons may be as an enhancer/mediator in the induction phase of CHA-induced hypothermia, but also function as a compensator during the recovery phase.

### Peripheral adenosine A1 receptor agonist-induced hypothermia was similar between ORX-AB and WT mice

Although orexin neurons are exclusively located in the central nervous system^17,21,22^, adenosine A1 receptors are not restricted to the brain^23^. Therefore, we next explored the possible effect of systemic administration of CHA on body temperature.

**Figure 4:**
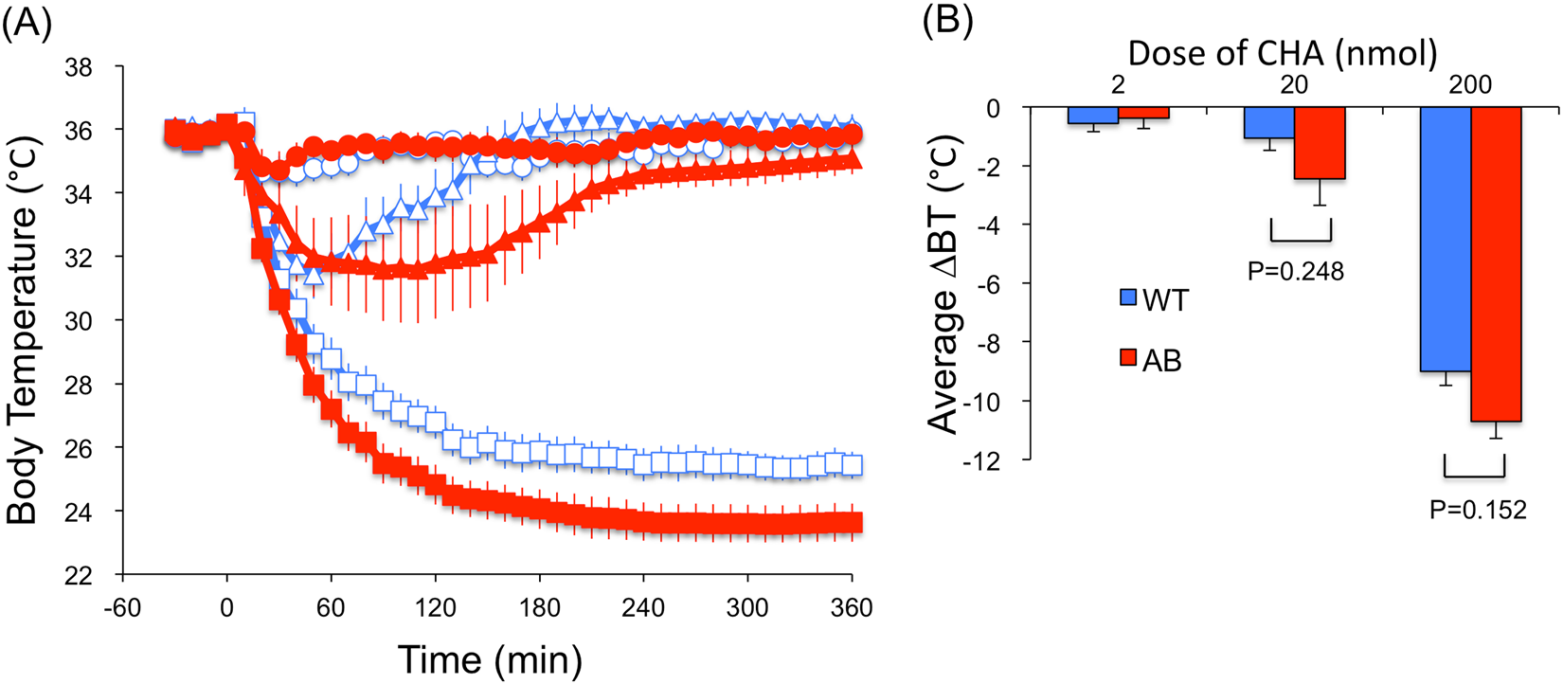
Effect of peripheral administration of an adenosine A1 receptor agonist, N6-cyclohexyladenosine (CHA), on body temperature. (**A**) Time-related changes in body temperature. Open symbols represent the data from wild-type mice (WT) and closed symbols from orexin neuron-ablated mice (AB). Circle, triangle, and square symbols indicate 2, 20, 200 nmol of CHA, respectively. CHA was intraperitoneally injected at time 0. (**B**) Changes in body temperature (BT) averaged for 6 hours after the injection. 2-way ANOVA indicated there was statistical difference among doses (F_2,30_ = 160.0, p < 0.0001) but not between genotypes (F_1,30_ = 4.11, p = 0.052). P values were calculated using the Holm-Sidak multiple comparison test. Each point represents mean ± SEM from 6 animals.

In both WT and ORX-AB mice, I.P. administration of CHA-induced hypothermia at doses of 20–200 nmol/mouse (Fig. 4). Average changes in body temperature during 6 hr tended to be greater in ORX-AB mice than in WT mice, though the difference did not reach statistical significance (F_1,30_ = 4.11, p = 0.052, by two way ANOVA). In contrast to I.C.V. injection (Fig. 3), there was no apparent difference in effective doses of CHA for either ORX-AB or WT mice with I.P. injection, indicating that the mechanisms of hypothermia were different depending on injection location. In addition, the doses required to cause hypothermia for I.P. injected CHA were ~100 (ORX-AB) to ~5,000 (WT) times higher than those in I.C.V. injection, indicating that the effect of I.C.V. CHA was not caused by spill out of the drug into systemic circulation.

When the results of both the I.C.V. and I.P. experiments are taken together, the possible role for orexin neurons in CHA-induced hypothermia seemed to be elicited only when central adenosine A1 receptors had been stimulated.

### Activation of orexin neurons during central adenosine A1 receptor agonist-induced hypothermia

Aforementioned results indicated the possibility that orexin neurons may accelerate recovery of body temperature when decreased (e.g. during fasting or resulting from central injection of CHA). To test this possibility, we next examined whether the orexin neurons were activated during hypothermia induced by central injection of CHA. We also examined possible changes in the activity of MCH neurons in both WT and ORX-AB mice.

Three hours after I.C.V. injection of either vehicle or CHA, the animals were deeply anesthetized and their brains were sampled. We selected a 3 hr time point because the nadir for body temperature was reached at around 2 hr after injection of CHA (Fig. 3A). Two doses of CHA were tested in both WT mice (0 and 0.02 nmol) and ORX-AB mice (0.02 and 0.2 nmol). In both animals, the lower dose induced negligible change in body temperature whereas the higher dose decreased the body temperature to be ~30 °C (Fig. 3A).

**Figure 5:**
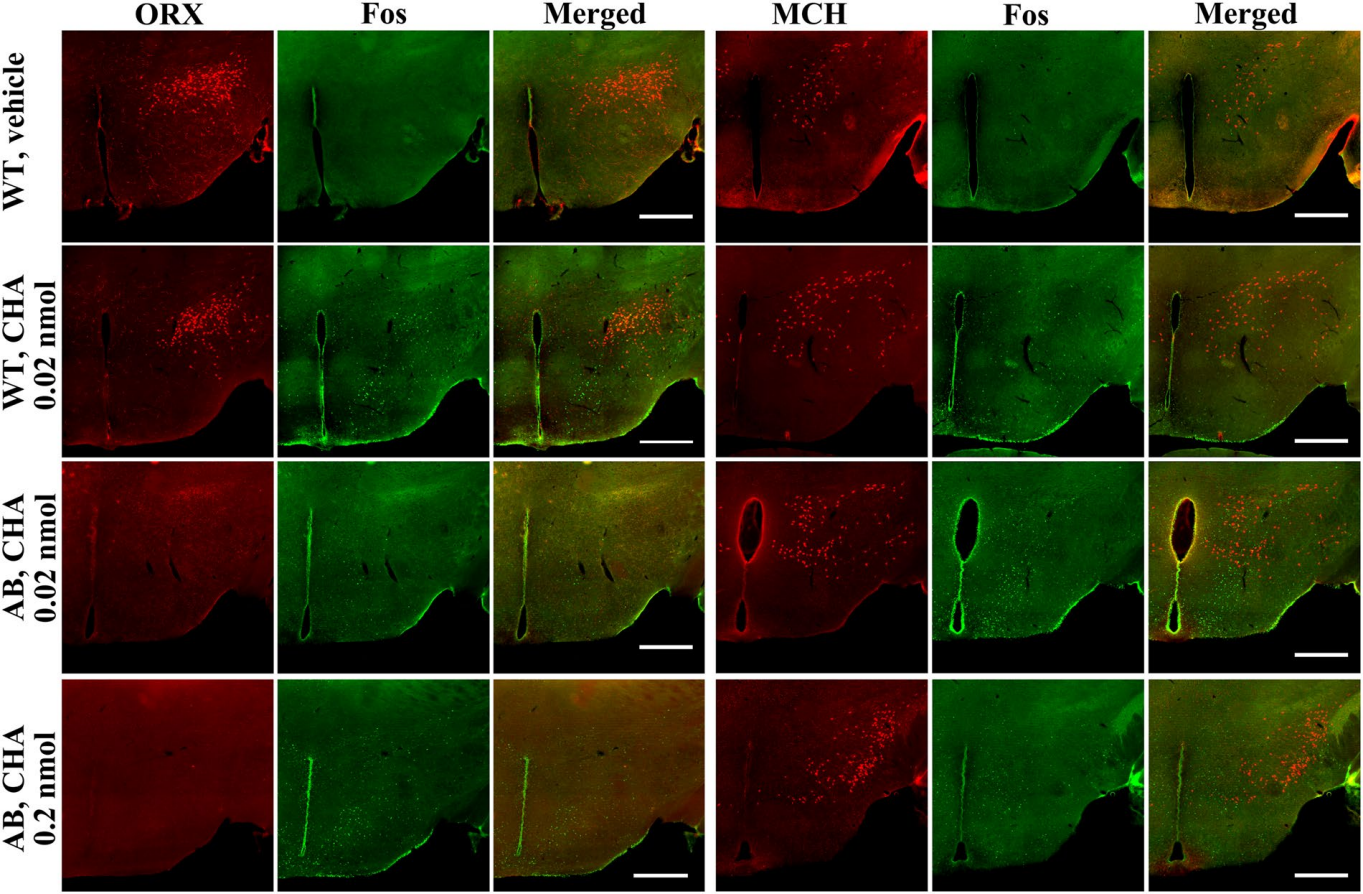
Effect of central administration of CHA on the hypothalamic neurons. Representative photographs show cellular activation marker c-Fos (green), orexin (ORX) or melanin concentrating hormone (MCH) immunoreactivity (red), and merged in the hypothalamus of vehicle-injected WT mice (top row), 0.02 nmol of CHA-injected WT mice (second row), 0.02 nmol of CHA-injected ORX-AB mice (third row), and 0.2 nmol of CHA-injected ORX-AB mice (bottom row). Horizontal bar indicates 500 µm. Note absence of orexin-immunoreactivity in ORX-AB mice (left end column, bottom 2 rows).

**Figure 6:**
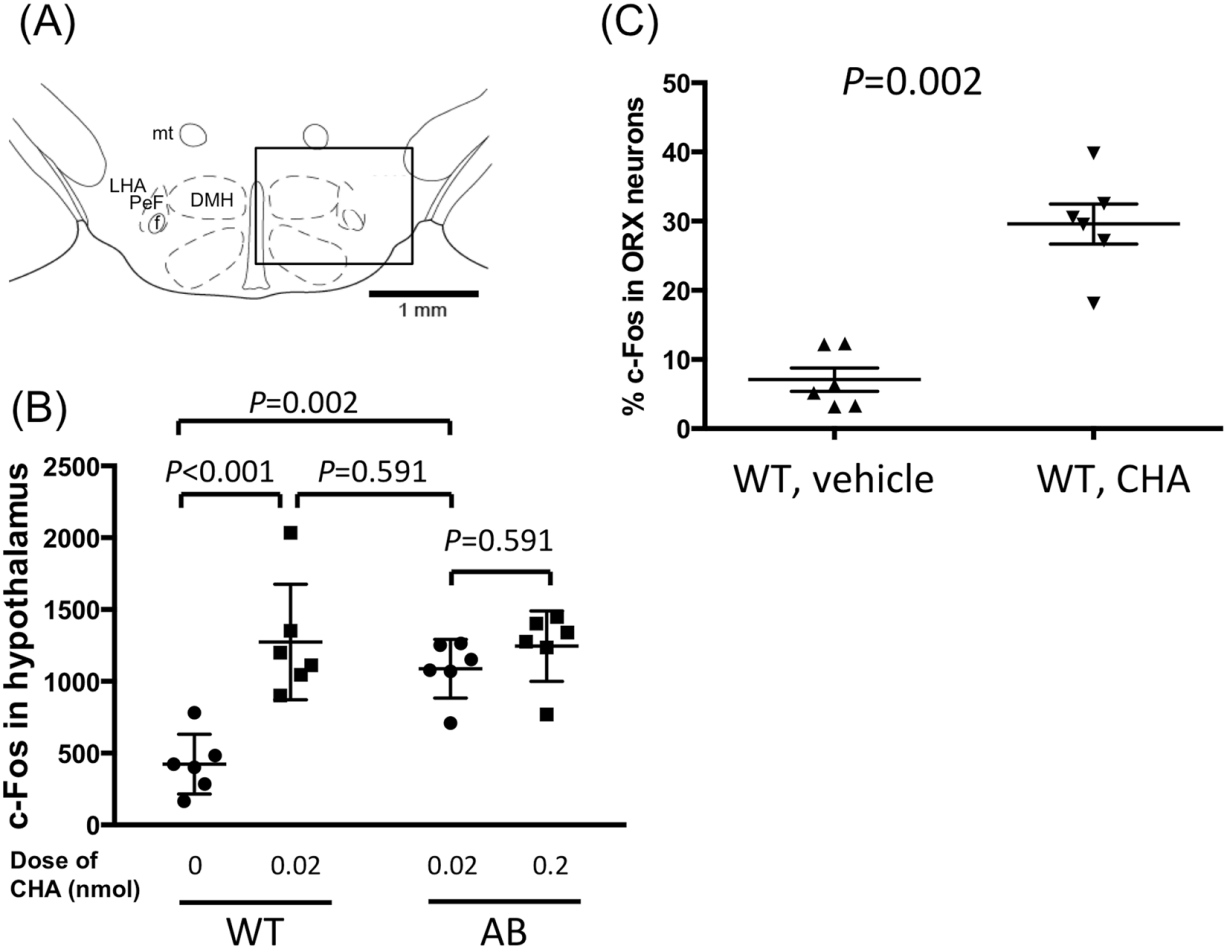
Activation of orexin neurons by CHA. (**A**) Square field denotes counting area for immunoreactivity. (**B**) The number of c-Fos positive cells among 4 groups of the mice: WT mice treated with vehicle, WT mice treated with 0.02 nmol of CHA, ORX-AB mice treated with low dose (0.02 nmol) of CHA, and ORX-AB mice treated with high dose (0.2 nmol) of CHA. 2-way ANOVA indicated there was statistical difference between doses (F_1,20_ = 7.89, p < 0.0108) and between genotypes (F_1,20_ = 19.86, p = 0.0002). P values were calculated using the Holm-Sidak multiple comparison test. (**C**) Percentage of the number of double positive cells among orexin immuno-positive cells. P value was calculated using the Mann-Whitney U-test. In (**B**) and (**C**), horizontal lines indicate mean and SEM of 6 animals. Keys for the brain structures: DMH, dorsomedial hypothalamus; f, fornix; LHA, lateral hypothalamic area; mt, mammillothalamic tract; PeF, perifornical area.

Immunohistochemical examination revealed ~900 orexin neurons in WT mice (917 ± 32 in ACSF group and 899 ± 55 in CHA group, n = 6 each) but did not in ORX-AB mice, as expected (Fig. 5). Similar numbers (~1,100) of MCH neurons were detected in the hypothalamus among 4 groups (1,111 ± 50 in the WT-ACSF group, 1,141 ± 62 in the WT-CHA group, 1,163 ± 66 in the ORX-AB-low dose CHA group, and 1099 ± 37 in the ORX-AB-high dose CHA group, n = 6 each) (Fig. 5). The number of c-Fos positive cells in the WT-CHA group (1274 ± 164) was significantly larger than in the WT-ACSF group (424 ± 85, p < 0.001) (Fig. 6B). The number of c-Fos positive cells in the ORX-AB-low dose CHA group (1088 ± 83) was not different from that in the ORX-AB-high dose CHA group (1245 ± 100, p = 0.591) and similar to that in the WT-CHA group (p = 0.591), indicating that the number of c-Fos positive cells in the hypothalamus may be related to CHA dosing but not to body temperature (note that low dose of CHA in ORX-AB mice did not decrease body temperature, Fig. 3).

The number of double positive cells for c-Fos and orexin in the WT-CHA group (264 ± 25, 29.6% of orexin cells) was significantly larger than in the WT-ACSF group (67 ± 17, 7.1% of orexin cells, p = 0.002) (Fig. 6C). The number of double positive cells for c-Fos and MCH was very low in WT-ACSF (8 ± 3, 0.8% of MCH cells), WT-CHA (10 ± 2, 0.9% of MCH cells), ORX-AB-low dose CHA (11 ± 2, 1.0% of MCH cells), and ORX-AB-high dose CHA (12 ± 3, 1.1% of MCH cells) groups. There was no significant difference among 4 groups.

These data clearly show activation of orexin, but not MCH, neurons at least during the recovery phase of CHA-induced hypothermia.

## Discussion

In this study, we examined the possible contributions of orexin neurons to fasting- and adenosine-induced hypothermia and achieved several interesting results. First, during 24 hr of food deprivation, the decrease in body temperature and the length of time suffering from hypothermia were greater in ORX-AB mice than those in WT mice, indicating a protective role for orexin neurons in fasting-induced hypothermia. Second, orexin neuronal activity was higher during the period preceding the hypothermic episode as well as during the recovery period towards the end of hypothermia. Third, when an adenosine A1 receptor agonist, CHA, was injected into the cerebral ventricle at doses of 0.02 and 0.04 nmol, WT mice experienced hypothermia as expected, whereas the body temperature of ORX-AB mice barely decreased, if at all. Higher doses of 0.2 and 2 nmol were required to induce hypothermia in ORX-AB mice. These results indicate that orexin neurons might play an inductive role in adenosine-induced hypothermia. Fourth, systemic injection of CHA resulted in similar decreases in body temperature as those observed in intracerebroventricular injection, but required much higher doses in both ORX-AB and WT mice. Taken together, the observed differences between ORX-AB and WT mice in central CHA-induced hypothermia seemed to be mediated by a central, but not peripheral, mechanism. Finally, central injection of CHA induced significant activation of the orexin neurons as revealed by c-Fos immunoreactivity. These observations support the notion that orexin neurons play a protective/compensative role in CHA-induced hypothermia at least during recovery period from hyperthermia.

### Orexin neurons protect from exaggeration of hypothermia and promote recovery from hypothermia

The most apparent difference between ORX-AB and WT mice in fasting-induced hypothermia was their body temperature at the nadir (29.0 ± 0.7 °C in ORX-AB vs. 32.3 ± 0.2 in WT mice) (Fig. 1). This observation implied two possibilities. First, orexin neurons may contribute in determining a set-point for body temperature during fasting. This notion is possibly supported by anatomical reports^17,21,22^ that show an orexin neuronal connection to the preoptic area, a presumed thermoregulatory center of the brain^24,25^. However, it is difficult to estimate a set-point from the present study because the ambient temperature was set at a fixed value of 21 ± 1 °C. Second, the possibility exists that orexin neurons will be activated compensatorily by the hypothermia and thus prevent a further decrease in body temperature. This notion explains the prolonged recovery from both fasting-induced (Fig. 1) and CHA-induced (Fig. 3) hypothermia in ORX-AB mice. In support of this idea, we showed that orexin neurons were activated during the recovery period of both fasting-induced (Fig. 2) and CHA-induced hypothermia in WT mice (Fig. 6). This notion also coincides with our previous observations: ORX-AB mice could not tolerate environmental cooling to 5 °C and their body temperature rapidly decreased below 30 °C within 1 to 2.5 hr whereas the body temperature of WT controls never decreased below 33 °C^12^. Also, ORX-AB mice showed exaggerated hypothermia and a prolonged recovery from isoflurane anesthesia^13^. The compensatory actions of orexin neurons on body temperature most likely took place through activation of the sympathetic nervous system controlling the brown adipose tissue^11,12^ which in turn controls the thermogenesis that plays a principal role in body temperature recovery from hibernation’s first phase^26^. The sympathetic nerves controlling the brown adipose tissue have been activated by local administration of orexin peptide into the medullary raphe^27^, where sympathetic premotor neurons controlling brown adipose tissues are located^28^. Orexin neuronal activity has been shown to be inhibited by glucose^14^ and hyperthermia^16^ *in vitro*. This literature collectively supports our notion. Therefore, we propose that orexin neurons play a protective role against hypothermia by limiting the decrease in body temperature and accelerating recovery probably through sympathetic activation.

### Orexin neurons paradoxically promote CHA-induced hypothermia

We originally expected that CHA-induced hypothermia would be exaggerated and prolonged in ORX-AB mice, as was the case in fasting-induced hypothermia. Contrary to our expectation, however, low doses of CHA (0.02–0.04 nmol) did not induce hypothermia in ORX-AB mice while doing so in WT mice (Fig. 3). At 0.02 nmol of CHA, a similar number of c-Fos positive cells was observed in the hypothalamus of ORX-AB mice to that of WT mice (Fig. 6B) indicating normal drug delivery and normal hypothalamus except orexin neurons in ORX-AB mice. Once hypothermia was induced at the higher doses of CHA (0.2–2 nmol), a large and long-lasting hypothermia was observed in ORX-AB mice. However, direct comparison for the higher doses CHA could not be completed because WT mice were unable to tolerate them. Thus, what we can convincingly conclude from this experiment was that ORX-AB mice had a lower sensitivity to CHA than WT mice. In other words, orexin neurons seemed to play an enhancing role in the induction of CHA-induced hypothermia.

Two opposite explanations may explain this apparent functional paradox. First, inhibition of orexin neurons via adenosine A1 receptor activation may be responsible for induction of CHA-induced hypothermia at lower doses of CHA. At least *in vitro*, the activity of orexin neurons has been shown to be inhibited by the adenosine A1 receptor^15^. However, the basal activity of orexin neurons does not seem to be the sole determinant of body temperature because the basal temperature of ORX-AB mice is not different from WT mice. In addition, intracerebroventricular administration of an antagonist for the orexin-A receptor, SB334867, has been reported to increase but not decrease the temperature of brown adipose tissue in the rat^29^. Therefore, the first possible explanation seems unlikely. The second explanation is that transient activation of orexin neurons and sympathetic activation may be responsible for induction of CHA-induced hypothermia. This explanation may seem odd, but some brain regions, including the dorsomedial hypothalamus where orexin neurons are located, have been shown to be activated during the early phase of hibernation in the golden hamster^30^. In addition, Swoap *et al*.^31^ reported that activation of the sympathetic nervous system controlling white adipose tissue and a decrease in circulating leptin were required to induce torpor in mice by using dopamine beta hydroxylase knockout mice which lack the sympathetic neurotransmitter noradrenaline. In line with this report, ob/ob mice lacking leptin suffer an increase in the number of torpors^32–34^. A decrease in leptin indicates an exhausted fuel storage and may be key for entering torpor. The finding that orexin neurons regulate the sympathetic nervous system controlling both brown and white adipose tissue^35^ coincides with our findings in regards to the mechanism. In addition, fasting-induced hypothermia was always preceded by transient increases in movement, heart rate, body temperature and, most importantly, orexin neuronal activity (Figs 1D and 2). Therefore, transient activation of orexin neurons seems to be a common phenomenon for the induction phases of both fasting- and CHA-induced hypothermia.

### Possible relationship between adenosine A1 receptor and orexin neurons

Although orexin neurons express adenosine A1 receptors^36^ and the direct action of adenosine on the neuron may be inhibition^15^, adenosine A1 receptors are also distributed in other neurons and thus intracerebroventricular administration of CHA can also indirectly affect the activity of orexin neurons. For example, neurons in the preoptic area (thermoregulatory center) and the nucleus of the solitary tract (the primary termination site of visceral afferents controlling gustatory and metabolic functions) express adenosine A1 receptors, and local injection of CHA into these areas has been shown to induce hypothermia^10,37^. If we had been able to specifically activate adenosine A1 receptors on orexin neurons, though out of scope of the present study, we would be able to make a conclusive remark on this issue.

### Fasting-induced vs. CHA-induced hypothermia

Adenosine, a direct metabolite of ATP, has been implicated as a signal of metabolic state and thus a key molecule required for entering torpor^38^. However, recent reports show a more complicated mechanism than has been presumed. Namely, mice lacking the adenosine A1 receptors and those lacking both the adenosine A1 and A3 receptors^39^ still showed fasting-induced hypothermia, but not CHA-induced hypothermia. Therefore, regulated hypothermia or torpor, can be induced by multiple brain mechanisms^39^. This study shows different contributions for orexin neurons in fasting- and CHA-induced hypothermia in its initial falling phase (inhibition in fasting-induced hypothermia and acceleration in CHA-induced hypothermia), and supports the idea that multiple mechanisms are involved in the induction of torpor. At the same time, orexin neurons probably contribute to recovery regardless of the source of the hypothermia (intrinsic or environmental).

In conclusion, orexin neurons seem to play dual roles (enhancer in the induction phase and compensator during the recovery phase) in adenosine-induced hypothermia and a protective/compensatory role in fasting-induced hypothermia. The results of the present study imply that there are multiple brain mechanisms involved in the induction of hypothermia but recovery relies, at least in part, on orexinergic activation.

## Methods

### Animals

All experimental procedures were performed in accordance with the guiding principles for the care and use of animals in the field of physiological sciences published by the Physiological Society of Japan (2015) and approved by the Institutional Animal Use Committees at Kagoshima University (MD14018, MD15016 and MD15075). All mice were housed in a room that was maintained at 22–24 °C and with lights on at 07:00 and off at 19:00 h. Mice had food and tap water available *ad libitum* unless otherwise indicated. All of the animal experiments were performed in a quiet and air-conditioned (21 ± 1 °C) room using 4–6-month-old male mice.

### Orexin neuron ablated mice

A method for selective ablation of orexin neurons has previously been reported^40^. In short, orexin-tTA mice, which express tetracycline transactivator (tTA) exclusively in orexin neurons under the control of the human prepro-orexin promoter^41^, were bred with tetO diphtheria toxin A fragment (DTA) mice (B6.Cg-Tg (tetO DTA) 1Gfi/J, The Jackson Laboratory) to generate orexin-tTA; tetO DTA mice. In these double transgenic mice (called ORX-AB in this paper), almost all (>97%) of the orexin neurons were ablated by 4 months of age^40^. Ablation of orexin neurons was confirmed in this study (see Fig. 5).

### Telemetric measurement of body temperature during fasting-induced hypothermia

Using a telemetric system (Dataquest, Data Sciences International, St Paul MN, USA), we measured abdominal temperature in freely moving mice in their home cages. At least 7 days before the experiment, a telemetric device (TA11TA-F10, Data Sciences International) was implanted in the abdominal cavities of mice while they were under anesthesia with 2–3% isoflurane. An antibiotic (penicillin, 40,000 U kg^**−**1^) and an analgesic (buprenorphine, 0.05 mg kg^**−**1^) were subcutaneously injected.

During the experiment, the mice were allowed access to food for one day, deprived of food for the next 24 h (19:00–19:00), and again supplied for the following day. Water was freely available for all three days. A pyro-electric passive infrared sensor was attached to the ceiling of the home cage to detect the animal’s movement^42^. Sensitivity of the movement sensor was set so that shivering would not be counted. Hypothermic episodes (torpor) were defined by a body temperature decrease below the minimum value obtained during the first day when the mice had full access to food. Body temperature was continuously monitored and digitized by PowerLab (ADInstruments, Castle Hill, Australia) and was stored and analyzed using LabChart software (ADInstruments).

### *In vivo* recordings of neuronal activity using fiber photometry

An improved version of the fiber photometry system (Lucir, Tsukuba, Japan) was used to record the activity of orexin neurons in conscious mice for a long period of time (>hr). The original system^43^ utilized only blue light (470 nm) excitation and photomultiplier detection (emission filter 525 ± 25 nm) for detection of G-CaMP6 fluorescence. In the current version, an additional excitation (590 nm) and emission (641 ± 75 nm) system was attached for detection of mCherry fluorescence, which is not affected by neuronal activity and thus can be used as an indicator of total stability of the fiber photometry system.

At least 3 weeks before the recording, a mixture of adeno-associated virus (AAV) vectors (serotype DJ) carrying tetO (3 G) G-CaMP6 and tetO (3 G) mCherry was stereotaxically injected into the hypothalamus of isoflurane (2–3%)-anesthetized orexin-tTA mice^43^. At the same time of AAV injection under isoflurane-anesthesia, a telemetric device (TA11ETA-F10, Data Sciences International) for recording body temperature and electrocardiogram was implanted in the abdominal cavities of mice^42^.

### Measurement of body temperature during adenosine A1 receptor agonist-induced hypothermia

Abdominal temperature was measured using a wireless passive transponder system (Electric Laboratory Animal Monitoring System, BioMedic Data Systems, Inc., Seaford DE, USA). Because the transponder (IPTT-300) is smaller (14 mm × 2 mm × 2 mm vs. 20 mm × 12 mm × 5 mm) and lighter (0.12 g vs. 1.6 g) than the telemetric device (TA11TA-F10), it may be more suitable for animal husbandry. However, this system is not suitable for long duration measurement (e.g. >24 h) because the experimenter must hold the scanner of the system (DAS7007R) within 5 cm of the transponder. In addition, approaching the scanner nearby the mouse may cause a stress. Care was taken that the mice were approached with the scanner from the rear in an attempt to avoid or minimize visual stimulation. In a preliminary experiment, we found that the data from the transponder system and the telemetric system were not significantly different (not shown) indicating any possible stress caused by the required close proximity of the scanner to the animal seemed negligible.

At least 7 days before the experiment, the transponder was implanted in the abdominal cavities of mice under isoflurane anesthesia. Also implanted was a guide cannula (C315GS-5/2.5; Plastics One Inc., Roanoke, VA, USA) for the intracerebroventricular administration of drugs to the lateral ventricle (1 mm lateral to the Bregma, 2.5 mm deep in the skull)^12^. The guide cannula was closed with a cannula dummy cap and firmly fixed to the skull with dental cement. Antibiotic and analgesic were injected as described above.

An adenosine A1 receptor agonist, N6-cyclohexyladenosine (CHA; Sigma-Aldrich) was dissolved in physiological saline to a concentration of 5 mM. It was diluted with artificial cerebrospinal fluid (ACSF) or saline to appropriate concentration on the day of experiment. Injection volume was 2 µl/mouse in the I.C.V. experiment and 0.3 ml/mouse in the I.P. experiment. Each animal received only one dose of the drug during the resting period (8:00–12:00). In this experiment, food was deprived from the recording chamber to avoid possible effect of adenosine A1 receptor agonist on food consumption^44^ that may secondary affect the body temperature.

### Immunohistochemistry

The activity of neurons producing orexin and melanin concentrating hormone (MCH) was assessed by double immunohistochemical staining for orexin/MCH and c-Fos using methods that were similar to those previously reported^12,45^. In brief, 3 h after the CHA or ACSF injections, the mice were deeply anesthetized by an I.P. injection of urethane (1.6 g kg^**−**1^) and transcardially perfused with 10 mM PBS, followed by a fixative solution containing 4% paraformaldehyde in PBS. Brains were excised and post fixed in the same fixative solution at 4**°**C overnight. Coronal sections, including the hypothalamus, were cut at 40 µm thickness using a vibratome. Every fourth section was collected, and free-floating immunohistochemical staining was performed. Sections were sequentially incubated with PBS containing 0.3% Triton-X and 2% normal horse serum for 30 min, rabbit anti-c-Fos antiserum (1:1000, ABE457, Merck Millipore, KGaA, Darmstadt, Germany) overnight, biotinylated donkey anti-rabbit IgG antibody (1:300, Jackson ImmunoResearch, West Grove, PA, USA) for 3 h, and goat anti-orexin antiserum (1:100, sc-8070, Santa Cruz Biotechnology, Inc., Santa Cruz, CA, USA) or goat anti-MCH antiserum (1:100, sc-14507, Santa Cruz Biotechnology) for 2 h at room temperature. Finally, the tissue was incubated with Alexa Fluor 488 streptavidin conjugate (1:500, Invitrogen Corp., Carlsbad, CA, USA) and CF568-labelled donkey anti-goat IgG antibody (1:500, Biotium, Heyward, CA, USA) for 90 min in a dark box. The sections were then mounted on a glass slide and examined with a fluorescence microscope (BZ-X700, Keyence Corp., Osaka, Japan). The number of orexin, MCH, and c-Fos positive cells were counted for both sides of five stained sections through the hypothalamus in a manner blinded to the treatment (CHA or ACSF).

To confirm AAV-induced expression of G-CaMP6 and mCherry in orexin neurons, orexin was visualized using CF647-labelled anti-goat IgG antibody (Biotium).

### Experimental Design and Statistical Analyses

#### Experiment 1

Fasting-induced hypothermia was examined in 6 ORX-AB mice and 6 WT control mice. Body temperature and movement were compared between the genotypes. In addition, onset latency, duration, and numbers of hypothermic episodes were compared. Comparisons were also made between fed and fasting and between day and night.

#### Experiment 2

Orexin neuronal activity around the fasting-induced hypothermia was measured in 5 mutant mice exclusively expressing G-CaMP and mCherry in the orexin neurons. Body temperature, heart rate, and fluorescence of G-CaMP and mCherry were compared among typical time points around hypothermic episode.

#### Experiment 3

Effect of I.C.V. injection of CHA on body temperature was examined in 36 ORX-AB mice and 24 WT control mice. Comparison was made among doses and genotypes.

#### Experiment 4

Effect of I.P. injection of CHA on body temperature was examined in 18 ORX-AB mice and 18 WT control mice. Comparison was made among doses and genotypes.

#### Experiment 5

Effect of I.C.V. injection of CHA on hypothalamic neuronal activation was examined in 12 WT mice and 12 ORX-AB mice using immunohistochemistry. Comparison was made among 4 groups (genotypes × low or high doses of CHA).

### Statistics

The Mann-Whitney U-test was used for comparison between the data from WT and ORX-AB mice. When necessary, a two-way ANOVA (genotypes × doses or genotypes × times) with post-hoc Holm-Sidak multiple comparison test was also used. Both were performed using Prism6 software (GraphPad Software, Inc.). The criterion for statistical significance was p < 0.05 in all cases. Data are presented as mean ± SEM.

The datasets generated during and/or analyzed during the current study are available from the corresponding author on reasonable request.
